# Impact of multicomponent home-based exercise on fear of falling in older people with a history of falls: insights from the GAITCARE project

**DOI:** 10.3389/fragi.2025.1698917

**Published:** 2025-12-10

**Authors:** Elisabet Huertas-Hoyas, Marta Neira Álvarez, Agustín Curiel-Regueros, Luisa Ruiz-Ruiz, Rafael García-Molina, Cristina Alonso Bouzón, Eva Rincon-Herrera, Sara García De Villa, Antonio R. Jiménez-Ruiz

**Affiliations:** 1 Physical Therapy, Occupational Therapy, Rehabilitation, and Physical Medicine Department, Rey Juan Carlos University, Madrid, Spain; 2 Department of Geriatrics, Foundation for Research and Biomedical Innovation of the Infanta Leonor University Hospital (FIIB HUIL), Madrid, Spain; 3 Universidad de Nebrija, Campus de Ciencias de la Vida, La Berzosa, Madrid, Spain; 4 Centre for Automation and Robotics, CSIC-UPM, Arganda del Rey, Madrid, Spain; 5 Department of Geriatrics, Perpetuo Socorro University Hospital, Albacete, Spain; 6 Department of Geriatrics, Foundation for Research and Biomedical Innovation of the Getafe University Hospital, Madrid, Spain; 7 School of Experimental Sciences and Technology, Rey Juan Carlos University, Madrid, Spain

**Keywords:** falls, fear of falling, exercise adherence, exercise digital applications, multicomponent exercise

## Abstract

**Aims:**

To compare the effectiveness of an 8-week multicomponent exercise program delivered at home with digital support versus conventional in-person hospital exercise sessions in reducing FOF.

**Materials and methods:**

The GAITCARE project is a multicenter quasi-experimental trial in three hospitals. Participants were assigned by hospital to either: (1) VIVIFIL App group—individualized daily home exercise with remote supervision; or (2) In person group—face to face exercise at hospital day-care units. The primary outcome was FOF measured by the Short Falls Efficacy Scale–International (Short FES-I). Secondary outcomes included adherence and app satisfaction.

**Results:**

127 participants were included (64 in App group, 63 in-person group), aged 70–93 (mean 82.36). FOF (SFES-I) was present in 68.3% of the in-person group and 54.7% of the App group. The 8-week intervention reduced FOF scores in both groups, reaching statistical significance only in the in-person group. However, the App group also showed a clinically relevant reduction (∼20%) despite starting with slightly lower baseline FOF, suggesting potential benefits of remote delivery. The in-person group showed higher adherence at weeks 4, 8, and 12 (follow-up). Baseline physical activity influenced adherence, with sedentary participants showing lower adherence. Digital delivery with remote supervision showed good feasibility and was generally well accepted by participants.

**Conclusion:**

FOF is prevalent in older adults with falls and can be significantly reduced by face-to-face group exercise, which also achieves higher adherence. Enhancements in telematic applications are necessary to improve adherence in digital interventions targeting FOF.

## 1 Introduction

Falls represent one of the leading causes of morbidity and mortality among older adults worldwide. Approximately 30% of people aged 65 years and older experience at least one fall each year, increasing to over 40% in those aged 80 and above (World Health Organization, 2021). Falls are also the main cause of injury-related hospital admissions and contribute substantially to healthcare and social costs (James et al., 2020).

An important contributor and consequence of falling is the fear of falling. Apart from physical complications, psychological consequences such as fear associated with falls might lead to a vicious cycle of inactivity, disability and more walking difficulties, increasing the risk of falls. Fear-related to falls was first reported as “post-fall syndrome” but this fear is no longer considered as a “post-fall syndrome” as more than 50% of the people with no prior fall experience fear of falling (FOF).

So, conceptually, there are two different approaches; the first one is “concern about falling (CAF)” focusing on the fearful anticipation of future falls and the second approach based on the construct “fall-related efficacy” defined as a low perceived self-efficacy in avoiding falls during daily activities (Tinetti et al., 1990) and assess by tools like Fall-Efficacy Scale (FES) by Tinetti et al. (1990) Probably they represent separate constructs that lie on a continuum and there are some new approaches to differentiate both of them (Takla et al., 2024) that will contribute to better understand their different impact in prevalence, consequences and intervention approach.

Fear of falling is common among older adults, with a global prevalence of 49.60%, ranging from 6.96% to 90.34%. Several risk factors have been identified for FOF, including advanced age, female sex, history of previous falls, living alone, chronic medical conditions, psychiatric disorders, neurological diseases, and frailty (Whitmore et al., 2024; Xiong et al., 2024).

Interventions that have demonstrated the greatest benefit in reducing FOF in community-dwelling older adults include physical exercise, although its effect is moderate and not sustained in the long term (Kendrick et al., 2014; Kumar et al., 2016), and cognitive therapy or meditation, which have shown modest results (Liu et al., 2018; Papadimitriou and Perry, 2020). Some studies suggest enhanced outcomes when both approaches are combined and delivered in-person under supervision (Kruisbrink et al., 2021; Sheng et al., 2024), possibly because supervised physical activity helps mitigate the anxiety, distress, and apprehension associated with engaging in exercise.

On the other hand, the integration of digital health technologies in geriatric care is increasing, particularly for fall monitoring and telehealth-based exercise promotion. These approaches may enhance accessibility for individuals with mobility limitations or those residing in areas with limited healthcare resources. In recent years, digital exercise programs have gained popularity as an alternative to conventional interventions; however, robust evidence on their effectiveness for reducing fear of falling remains scarce. Moreover, very few studies have directly compared in-person versus digital exercise delivery among very old and frail populations, who may face additional barriers to technology use and adherence.

Our research group has developed a tailored multicomponent exercise program for older adults with a history of falls. The program is individualized based on each participant’s functional reserve and is delivered through a mobile application (VIVIFIL App), which is specifically designed for older adults and enables remote supervision and monitoring by healthcare professionals giving the opportunity to strength relationship with the participant.

Hypothesis: The use of a mobile application specifically designed for older adults, incorporating remote supervision, will be as effective as in-person interventions in reducing fear of falling.

Primary objective: To compare the efficacy of an 8-week multicomponent exercise program delivered at home with digital support (VIVIFIL App) versus a hospital-based, group exercise program in reducing fear of falling, as measured by the Short Falls Efficacy Scale–International (Short FES-I).

Secondary objectives:

1. To evaluate adherence to both exercise programs.
2. To assess participant satisfaction and perceived usability of the digital application.

## 2 Materials and methods

### 2.1 Study design

**FIGURE 1:**
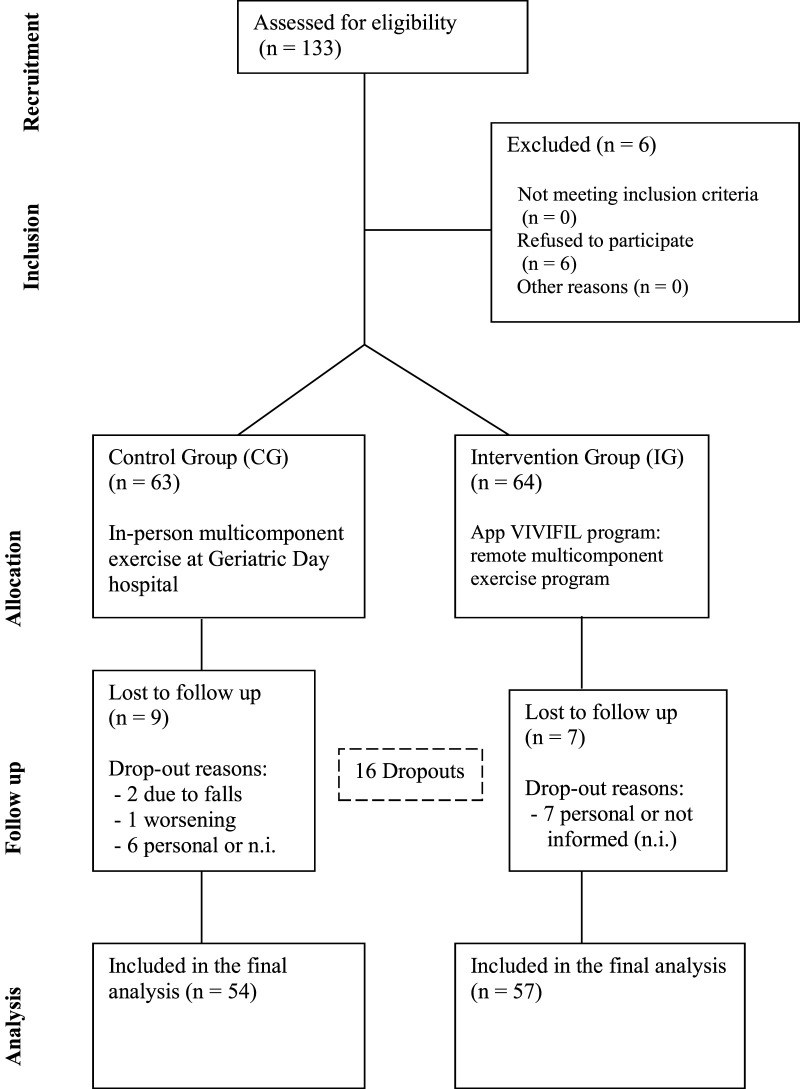
Consort flow diagram.

The GAITCARE project is a multicenter quasi-experimental study (non-randomized clinical trial) conducted in three Spanish public university hospitals. Participants were assigned to intervention groups according to the hospital in which they received care. Randomization was not feasible due to organizational and logistical constraints across centers, as the study was designed to integrate the intervention into standard clinical practice. The trial followed the CONSORT (Figure 1) extension guidelines for non-randomized studies and included an 8-week intervention period with follow-up assessments conducted at the end of the program.

The study was approved by the Research Ethics Committee on Medicinal Products (CEIm) of the Hospital Universitario de Albacete on 27 June 2023 (Reference code No. 2023-071) and has been retrospectively registered on ClinicalTrials.gov (identifier: NCT06936865).

### 2.2 Participants

Individuals aged 70 years or older with a history of falls in past 12 months were recruited from the specialized Fall Clinics of the three participating hospitals between December 2023 and March 2024.

Participants were recruited from Falls Clinics to which they were referred for evaluation by a geriatric specialist, where a comprehensive, multidimensional assessment was conducted. This referral pathway allows any physician in community or hospital settings to refer patients with a documented history of falls who meet guideline-defined fall-risk criteria (Montero-Odasso et al., 2022). Each of the three hospitals has a Falls Clinic and maintains a formal research collaboration on falls. All participants met the eligibility criteria and those from Albacete and Getafe hospital agreed to attend the intervention program at the Geriatric Day Hospital, and all participants provided written informed consent.

Inclusion criteria were based on these recommendations: adults aged 70 years or older; able to ambulate independently (with or without a mobility aid, but without requiring the physical assistance of another person); and presenting with at least one of the following: (1) one fall with consequences requiring medical attention within the past 12 months; (2) two or more falls in the past 12 months; (3) one fall plus self-reported gait/balance disturbances; or (4) one fall plus self-reported fear of falling. Exclusion criteria included a terminal illness with life expectancy <6 months; severe cognitive impairment; major sensory impairment preventing safe app use; or failure to provide informed consent.”

### 2.3 Study measures

#### 2.3.1 Primary outcome

Fear of Falling (FOF): It is assessed by the Short Falls Efficacy Scale - International (Short FES-I). This is the short version of the 7-item self-administered scale designed to assess fear of falling in older adults living primarily in the community. The validated Spanish version was used, and it was administered as an interview by a member of the research team. The 7 items include a variety of functional activities of daily living. The person is asked to score 1 if they are not worried about falling to 4 if they are very worried during different activities. The score ranges from a minimum of 7 (no concern) to 28 (severe concern). From 9 points upwards is considered moderate worry (Kempen et al., 2008) and ≥11 is considered cutoff point to define high fear of falling (Kumar et al., 2014; Scheffers-Barnhoorn et al., 2023).

#### 2.3.2 Secondary outcomes

1. Adherence to training activity: Adherence was measured according to attendance to training activity and was recorded daily during in-person training at the corresponding hospitals. In the digital remote group, the App tracks progress by registering when each exercise is completed, allowing the participant to move on to the next one. It is not necessary to complete the entire training session in a single sitting, and adherence is quantified based on each exercise performed rather than on finishing the full session. Adherence was defined as high when attendance was above 50% of the time program and low adherence when participants attended less than 50% of sessions. For adherence analysis, only participants who remained in the study and completed the final evaluation were included. Although >80% adherence is often used as the standard threshold, we adopted >50% considering the characteristics of our frail, oldest-old population. This decision is supported by a recent scoping review on the adoption and adherence of physical activity mobile applications by older adults (Lorenzi et al., 2024; Delbaere et al., 2021), which emphasizes that multiple personal and technical barriers affect long-term adherence in this population.
2. Satisfaction with App VIVIFIL: This was assessed at the end of the intervention (week 8) using a custom survey administered as an interview. It consisted of three items rated on a 5-point Likert scale (Strongly Disagree, Disagree, Don’t Know, Agree, Strongly Agree). The items evaluated: (1) perceived complexity of the app, (2) appropriateness of the exercises for the user’s functional level, and (3) global usefulness of the app The questions were: ¿Have you considered the App VIVIFIL complex to use or low intuitive? ¿Are exercises in VIVIFIL App appropriate for your activity and gait level?¿Globally, do you consider App VIVIFIL useful for you?
3. Sociodemographic characteristics: participants were asked aboutage, gender, education background, marital status and physical activity level (active/sedentary): men were considered sedentary if they walked less than 3 h per week or had an energy expenditure below 459.6 kcal and women were sedentary if they walked less than 1 h per week or had an energy expenditure below 135 kcal (Fried et al., 2001).
4. Clinical characteristics: Cognitive assessment using the Global Deterioration Scale (GDS) by Reisberg (Auer and Reisberg, 1997). Frailty (FRG) assessed using the Standardised Frailty Criteria (Alonso Bouzón et al., 2017). Physical performance measured by the Short Physical Performance Battery (SPPB) (Guralnik et al., 1994). Hand grip strength (HGS): measured using a JAMAR hydraulic hand dynamometer To allow for the expression of maximum voluntary force, the measurement was taken with the participant in a standing position and the elbow fully extended, following protocols from recent literature (Xu et al., 2021). Participants performed three grip strength trials with their dominant hand, and the highest value (in kilograms) was recorded (Xu et al., 2021).

### 2.4 Intervention procedure

The intervention program consisted of an 8-week multicomponent exercise regimen including aerobic, strength-power, flexibility, and balance exercises, delivered either remotely or in person, depending on the participant’s hospital and according to standard clinical practice:

### 2.5 The exercise prescription was tailored to the delivery modality

The App group from Hospital Universitario Infanta Leonor, which lacks a Geriatric Day Hospital, completed a home-based exercise daily program supported by the VIVIFIL mobile app and remotely monitored by a trainer. It was prescribed daily sessions of approximately 30 min, with a structure of five core days and two optional make-up days, aiming for a weekly goal of 150 min of exercise.

In contrast, the onsite participants from the Geriatrics Departments of Hospital Universitario de Getafe and Hospital Universitario de Albacete (control group) received conventional, in-person care at Geriatric Day Hospitals. This session frequency (2-4 times/week) was determined by the established protocols of each hospital’s Geriatric Day Hospital.

While both programs shared a multicomponent structure, specific exercises were adapted for the home environment in the App group. Both interventions were supervised by qualified staff, including therapeutics and sports scientists. Real-time corrective feedback was inherent to the onsite sessions, while for the App group, it was provided upon participant request via the integrated chat.

While the average weekly exercise time was similar across groups (approximately 150 min), the APP group completed more frequent (daily sessions) but shorter sessions, whereas the in-person group had fewer sessions of longer duration.

### 2.6 App VIVIFIL

The App, developed by the research team for Android and iOS, was co-designed with older adult volunteers and tailored to their needs. It enabled professionals to prescribe individualized exercise programs based on functional status (SPPB stratification), promoting daily autonomous training following a progressive model (i.e., increasing duration, sets, and repetitions). The app installation was conducted in-person during the baseline visit. Participants received a hands-on tutorial and were informed about the available support channels. Each exercise within the app was accompanied by a video demonstration and a voice-guided countdown. To ensure continuous assistance, a two-way chat with an assigned assistant and telephone support were provided to resolve any technical or exercise-related queries.

**FIGURE 2:**
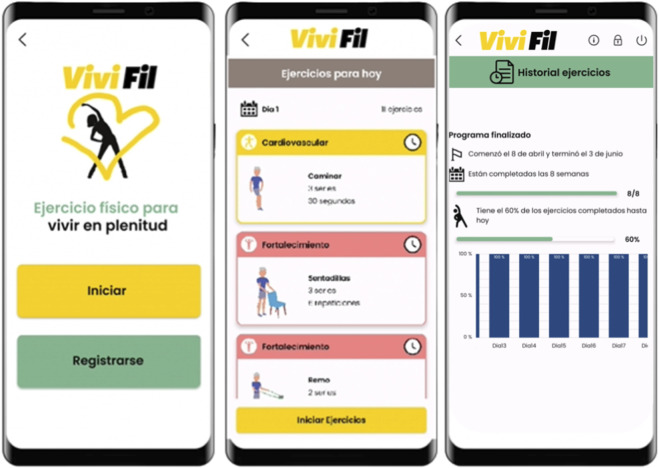
App VIVIFIFIL interface showing how exercise is presented and adherence recorded.

Particular attention was given to usability aspects to facilitate engagement in this population. The interface incorporated large buttons and font sizes to improve accessibility, and each exercise was accompanied by explanatory videos and schematic illustrations to ensure correct execution. A voice-guided countdown was also integrated to support exercise performance. Additionally, the App included a chat function for two-way communication, allowing participants to pose questions and receive support either through the App or by phone, thereby enhancing adherence and enabling remote follow-up (Figure 2). Each completed exercise was automatically registered within the app, allowing supervisors to monitor adherence and exercise frequency remotely. Monitoring was asynchronous; supervisors accessed performance records through the secure platform rather than observing participants in real time. Data collection and storage followed the privacy and security standards of the European General Data Protection Regulation (EU) 2016/679 (GDPR).

Unlike generic digital platforms such as WhatsApp, YouTube, or Zoom, which only allow passive exercise delivery or basic communication, the VIVIFIL App was specifically designed for older adults with functional limitations and risk of falls. It integrates professional prescription, individualized adaptation based on functional reserve, and automatic adherence monitoring within a single tool. Moreover, its interface and interaction model were co-designed with older users to ensure accessibility, simplicity, and engagement, addressing sensory and cognitive limitations common in this population. This approach fills a gap in existing digital health interventions, which often focus on general wellness rather than clinically guided, personalized exercise programs with remote supervision and real-time feedback.

### 2.7 Data colletion

Both groups underwent a baseline assessment at week 0, which included evaluation of all sociodemographic, cognitive, functional (FRG, SPPB) variables and fear of falling by SFES-I. Each participant was assigned a multicomponent exercise program based on their functional status (SPPB score) and allocated to an intervention group according to their hospital. In the VIVIFIL App group, the app was installed on participants’ personal devices if these were compatible. To ensure inclusivity, participants without a compatible or up-to-date smartphone were provided with a device for the study period, configured with free internet access. On the day of consent and device setup, all participants in this group received a comprehensive, in-person training session on using the smartphone and the VIVIFIL application. This session covered navigation, starting exercises, and utilizing the support chat function.

Over the 8-week intervention period, participants followed their assigned programs. At the end of the intervention (week 8), a final in-person assessment was conducted at the hospital for all participants. This session, which lasted approximately 40 min, and included SFES-I, adherence, satisfaction with app and functional aspects (FRG and SPPB). In the App group, correct exercise execution was monitored through weekly therapist review, during which therapists checked participants’ exercise performance and provided feedback through the App to ensure proper technique and safety. Additionally, there were two more telephonic visits to know adherence and satisfaction with App in weeks 4 and only satisfactioin in week 12. Outcome assessments were conducted by trained clinicians who were not blinded to group allocation. Standardized protocols were followed to ensure consistency and reduce measurement bias.

### 2.8 Data analysis

Statistical analysis was performed using SPSS software version 31 (2013 IBM SPSS Corp.) and MATLAB R2022a (The MathWorks, Inc., Natick, MA, United States) with the Statistics and Machine Learning Toolbox.

As the data did not follow a normal distribution, non-parametric tests were used. Quantitative variables were expressed as means and standard deviations, while qualitative variables were presented as percentages. Inter- and intra-group comparisons across the three time points were conducted using the Mann–Whitney U test (continuous variables) and the Chi-Square test or Fisher’s exact test when expected frequencies in any cell were below 5 (categorical variables). Intragroup comparisons between two time points (e.g., pre-post) were performed using the Wilcoxon signed-rank test, Friedman test when comparisons were between three points (pre, post and follow-up) and McNemar test for related categorical samples. Correlations between variables were assessed using Spearman’s rank correlation coefficient. In addition, a linear regression analysis was conducted to explore the influence of potential confounding variables on adherence levels. Analyses were performed on a per-protocol basis, including participants who completed all intervention and follow-up assessments.

The level of statistical significance was set at p < 0.05. Sample size estimation was performed using G*Power 3.1 software, based on the following parameters: Student’s t-test with a large effect size of 0.5 (Cohen, 1988), alpha error of 0.05, statistical power of 0.80 (Sun et al., 2021), two groups, and three measurements (pre-, post-, and follow-up). Based on these parameters, the estimated required sample size was 102 participants. Accounting for an anticipated 10% dropout rate, the final sample size for the study was set at 112 participants (56 per group).

## 3 Results

A total of 127 participants were included in the GAITCARE project, with 64 allocated to the VIVIFIL App group and 63 to the group receiving in-person exercise. There were 16 dropouts (12.59%) during the 8-week intervention period. In the App group, 7 participants withdrew for personal reasons and in the in-person group, 9 participants were discontinued (two due to falls with complications, one due to worsening of an underlying condition and six for personal reasons). Most of personal reasons were not informed.

Baseline characteristics of the total sample and both groups are presented in Table 1. The mean age of the sample was 82.36 years (SD = 5.53), ranging from 70 to 93 years and 66.9% were women. Most participants were sedentary, 60.63% were frail and 40.9% of total participants had no educational studies. Statistically significant differences between groups were observed in age, SPPB scores and mean FES-I performance.

**TABLE 1:** Baseline participant characteristics (Week 0).

Parameter	Total (N = 127)	Onsite (N = 63)	App (N = 64)	Sig
Age (years)	82.36 ± 5.53 [70–93]	80.78 ± 5.68 [70.00, 93.00]	83.92 ± 4.95 [73.00, 93.00]	0.002
Weight (kg)	67.45 ± 12.48 [38.1–105]	68.67 ± 13.1 [38.1–100.6]	66.25 ± 11.82 [45–105]	0.303
Height (m)	1.55 ± 0.08 [1.36, 1.76]	1.58 ± 0.09 [1.37, 1.76]	1.53 ± 0.07 [1.36, 1.72]	0.005
BMI	27.7 ± 4.41 [17.16, 41.34]	27.4 ± 4.51 [17.16, 41.34]	28.06 ± 4.33 [18.87, 40.74]	0.371
HGS	17.9 ± 7.51 [6.6, 44]	19.07 ± 8.07 [7.30, 44]	16.86 ± 6.8 [6.6, 35.50]	0.139
Sex	Men: 42 (33.1%)	Men: 23 (36.5%)	Men: 19 (29.7%)	0.41
	Women: 85 (66.9%)	Women: 40 (63.5%)	Women: 45 (70.3%)	​
Marital status				
married	57 (44.9%)	33 (52.4%)	24 (37.5%)	0.33
Widowed	65 (51.2%)	28 (44.4%)	37 (57.8%)	
Separated	2 (1.6)	1 (1.6%)	1 (1.6%)	
Single	3 (2.4%)	1 (1.6%)	2 (3.1%)	
Education level				
Primary educ	64 (50.4%)	37 (58.7%)	27 (42.2%)	0.11
Bachelor’s degree	11 (8.7)	6 (9.5%)	5 (7.8%)	
No educational	52 (40.9)	20 (31.7%)	32 (50%)	
FRG	Fit 5 (3.94%)	Fit: 3 (4.76%)	Fit: 2 (3.13%)	0.24
	Prefrail 45 (35.43%)	Prefrail: 18 (28.57%)	Prefrail: 27 (42.19%)	
	Frail 77 (60.63%)	Frail: 42 (66.67%)	Frail: 35 (54.69%)	
Physical activity	Active: 49 (38.6%)	Active: 24 (38.1%)	Active: 25 (39.1%)	1.14
	Sedentary: 78 (61.4%)	Sedentary: 39 (61.9%)	Sedentary: 39 (60.9%)	​
GDS (Index)	1.33 ± 0.68 [1–4]	1.43 ± 0.78 [1.00, 4.00]	1.23 ± 0.56 [1.00, 3.00]	0.170
SPPB (Index)	7.54 ± 2.78 [1–12]	8.21 ± 2.38 [2.00, 12.00]	6.88 ± 2.99 [1.00, 12.00]	0.011
SFES-I	12.94 ± 5.17 [7–28]	14.76 ± 5.68 [7–28]	11.27 ± 4.01 [7–25]	0.001
SFES-I ≥11 n (%)	78 (61.5%)	48 (68.3%)	35 (54.7%)	0.073
SFES-I ≥9 n (%)	91 (71.7%)	53 (76.3%)	43 (67.2%)	0.096

BMI, Body Mass Index; HGS, hand grip strength; FRG, Standardised frailty criteria; SPPB, Short physical performance battery; SFES-I, short falls efficacy scale–international.

### 3.1 Fear of falling: SFES-I

Table 2 presents the results reflecting the SFES-I measured at week 8, after completion of the intervention, in both groups.

**TABLE 2:** Comparison between independent samples according to SFES-I at week 8, n = 111.

Parameter	Onsite (N = 54)	App (N = 57)	U/Fisher’s test	Sig
SFES-I (mean, SD)	12.39 ± 4.48 [7, 28]	11.05 ± 3.8 [5, 24]	−1.50	0.132
SFES-I ≥11 n (%)	30 (47.7%)	27 (42.1%)	19.66	0.01
SFES-I ≥9 n (%)	42 (66.7%)	42 (65.6%)	21.95	0.02

SFES-I ≥ 11 = Short Falls Efficacy Scale – International with a score greater than or equal to 11 points (cut-off point). SFES-I ≥ 9 = Short Falls Efficacy Scale – International with a score greater than or equal to 9 points (cut-off point).

In the intervention group using the VIVFIL App, 42.1% of participants reported a very high fear of falling and 65.6% a moderate fear of falling at week 8, with no significant differences compared with baseline. Similarly, the mean SFES-I score showed no significant change, decreasing slightly from 11.27 ± 4.01 at baseline to 11.05 ± 3.8 after the intervention.

To examine potential associations between fear of falling and sociodemographic variables (age, sex, weight, height, BMI, marital status, education level, GDS score, physical activity, and HGS), correlation analyses were performed between baseline and post-intervention SFES-I scores and these variables. Our findings indicated a significant negative correlation between post-intervention SFES-I and physical activity (ρ = −0.331, *p* < 0.01), as well as significant positive correlations with FRG both at baseline (ρ = 0.420, *p* < 0.01) and post-intervention (ρ = 0.506, *p* < 0.001). A significant negative correlation was also observed with SPPB at baseline (ρ = −0.370, *p* = 0.003) and post-intervention (ρ = −0.386, *p* = 0.003).

To determine whether there was a relationship between fear of falling and sociodemographic variables (age, sex, weight, height, BMI, marital status, education, GDS, physical activity, and HGS), a correlation study was conducted between baseline and post-intervention SFES-I scores and these variables. The analysis showed a negative relationship between post-intervention SFES-I and physical activity level (ρ = −0.331, p < 0.01), FRG before the intervention (ρ = 0.420, p < 0.01) and after the intervention (ρ = 0.506, p < 0.001), as well as a negative correlation with SPPB before the intervention (ρ = −0.370, p = 0.003) and after the intervention (ρ = −0.386, p = 0.003).

In the in-person intervention group, 68.3% of participants reported a high fear of falling at baseline (week 0), which decreased significantly to 47.7% after the 8-week program. Correlation analyses showed significant associations between baseline SFES-I and sex (ρ = 0.399, *p* = 0.02), height (ρ = −0.417, *p* < 0.001), physical activity (ρ = −0.344, *p* = 0.008), and strength (ρ = −0.346, *p* = 0.007). Post-intervention SFES-I scores were also significantly correlated with sex (ρ = 0.463, *p* < 0.001), height (ρ = −0.404, *p* = 0.002), and strength (ρ = −0.367, *p* = 0.006).

**FIGURE 3:**
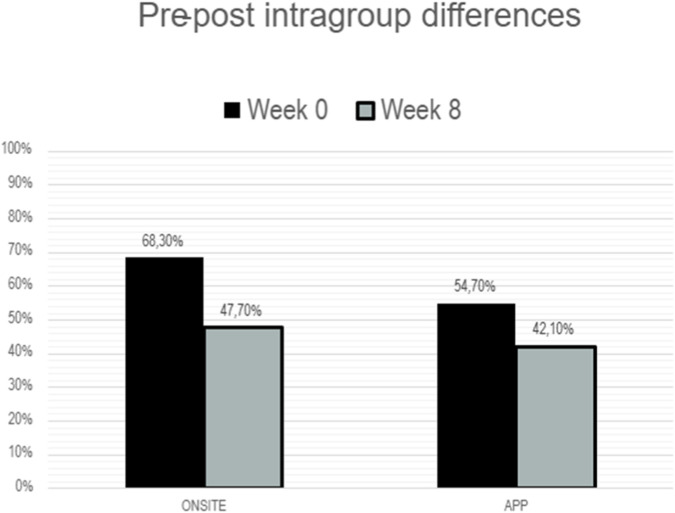
Frequencies on the SFES-I scale based on the cutoff point (≥11) for the Onsite and App modalities at weeks 0 and 8 (pre-vs. post-intervention).

Figure 3 presents the proportion of participants with SFES-I ≥ 11 at baseline and week 8 in both groups, illustrating the effect of the exercise-based intervention on fear of falling. Although differences were observed, the data show that these differences are not significant in the in-person group (p = 0.065), nor in the App group (p = 0.070).

### 3.2 Rate of adherence to the program

**FIGURE 4:**
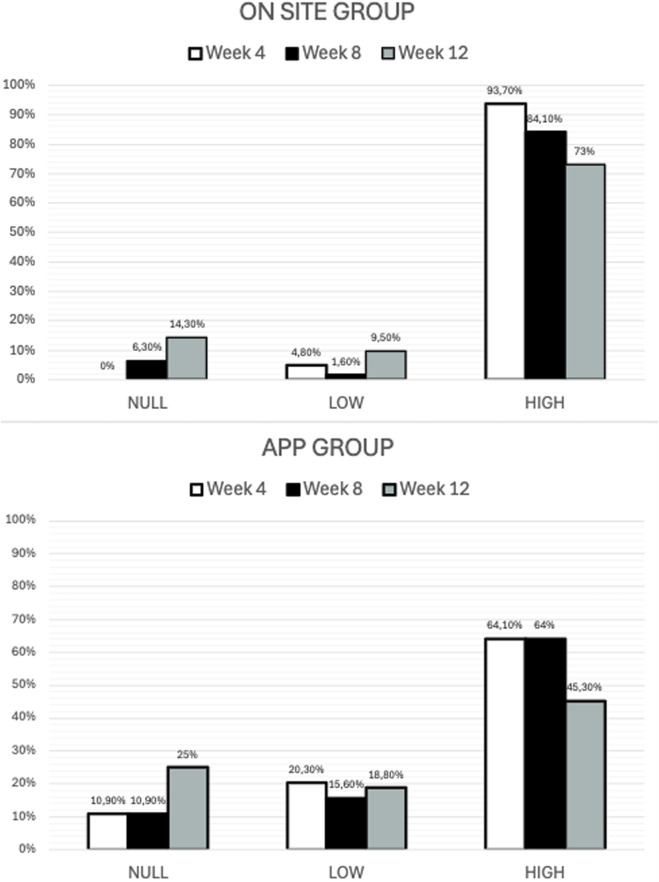
Distribution of Adherence Levels by Intervention Modality at week 4, 8 and 12 (follow-up) in the onsite group and in the App group.

Figure 4 illustrates the progression of adherence levels at weeks 4, 8, and 12 across the two intervention groups (onsite and App-based). In the onsite group, the proportion of participants with high adherence remained consistently elevated throughout the study period (93.7% at week 4, 84.1% at week 8, and 73% at week 12), while the proportions with low or null adherence were minimal. A Friedman test was conducted to compare adherence levels at three time points (baseline, post-intervention, and follow-up). Results revealed statistically significant differences over time, χ^2^ (2) = 36.14, *p* < 0.001. Adherence was highest at baseline, decreased slightly post-intervention (from 93.7% to 84.1%),and declined more substantially at week 12 (73%). These findings indicate that the intervention promoted high adherence, although a decline was observed once the active phase ended (Figure 4).

In contrast, in the App group, high adherence progressively decreased over time (64.1% at week 4, 64% at week 8, and 45.3% at week 12), accompanied by increases in low and null adherence. The Friedman test also demonstrated statistically significant differences in adherence across the three time points, χ^2^ (2) = 15.55, *p* < 0.001. Mean rank analyses indicated stable adherence from baseline to post-intervention, followed by a significant reduction at week 12 (from 64.1% to 45.3%). These findings suggest that onsite intervention was associated with greater and more sustained adherence compared with the App-based modality (Figure 4).

A confounder analysis was conducted to evaluate the influence of additional variables on adherence levels. The model was statistically significant, F (6, 118) = 3.22, *p* = 0.006, accounting for a significant proportion of variance in adherence (Table 3). Among the predictors, only physical activity level was significantly associated with exercise adherence (B = 0.651, β = 0.343, *p* < 0.001), indicating that higher baseline physical activity predicted higher adherence. Other variables (age, sex, marital status, education level, and GDS) were not significant predictors.

**TABLE 3:** Predictors on adherence level.

Predictor	R^2^ = 0.141, F (6,118) = 3.22, p = 0.006				
	B	SE	β	t	p
Age	−0.006	0.015	−0.039	−0.423	0.673
Sex	0.312	0.185	0.161	1.681	0.095
Marital status	−0.132	0.126	−0.094	−1.041	0.300
Education level	0.231	0.141	0.159	1.644	0.103
GDS	0.112	0.116	0.083	0.961	0.339
Physical activity	0.651	0.165	0.343	3.936	<0.001

### 3.3 Self-opinion about usability of the VIVIFIL app

**FIGURE 5:**
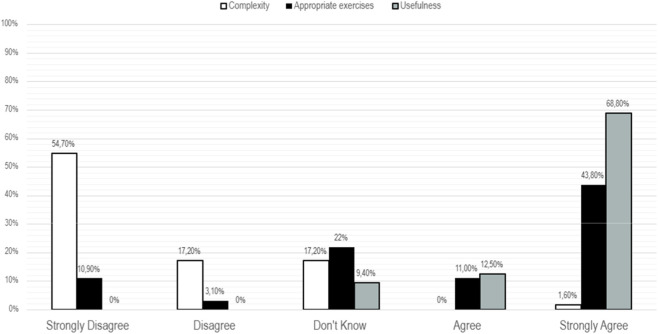
Perceptions of participants who used the App during treatment.

Regarding participants’ satisfaction with VIVIFIL App (Figure 5), data indicated that 54.7% of participants reported that the App was not complex to use, whereas 17.2% considered it complex, and only 1.6% strongly agreed that the App was complex.

A total of 43.8% of participants strongly agreed that the exercises proposed in the VIVIFIL App were appropriate, with an additional 12.5% agreeing and only 10.9% strongly disagreed, suggesting that the exercises were generally well rated.

Perceptions of overall usefulness were markedly favorable: 68.8% of participants strongly agreed that the content was useful, and an additional 11% agreed. No participants expressed disagreement with this statement, indicating a highly positive perception of the App’s usefulness.

## 4 Discussion

The study found that fear of falling (SFES-I) was more prevalent in the onsite group (68.3%) than in the app group (54.7%). After an 8-week multicomponent program, only the onsite group showed significant reductions in SFES-I, likely aided by slightly higher baseline FOF. On adherence, onsite participants had higher rates at weeks 4 and 8, though over 45% of app users still achieved high adherence (>50% completion). Baseline physical activity was the sole predictor of adherence, with sedentary individuals adhering least, suggesting onsite programs foster more sustained engagement and lower dropout.

FOF is a multifactorial and complex construct, and the SFES-I scale reflects subjective perceptions that may be influenced by contextual factors such as item phrasing, interviewer-participant rapport, educational and cultural background, emotional status (e.g., depressive or anxiety symptoms), previous fall experiences, and perceived social support. These elements likely contribute to the variability in reported prevalence across studies (Xiong et al., 2024; Tomita et al., 2016; Drootin, 2011; Ellmers et al., 2025; Machiko et al., 2016; Nicholson et al., 2024). Xiong et al. reported a global prevalence of 49.6% (range, 6.96%–90.34%), highlighting the influence of gender, educational, physical, and social factors and, consistent with our results, helping to explain the baseline differences observed in this trial. Although such differences limit strict comparability between groups, our analyses focused on within-group effects of the intervention.”

Regarding intervention strategies for FOF most reviews emphasize the low methodological quality and high risk of bias in existing studies, limiting the strength of available evidence. Meta-analyses (Kruisbrink et al., 2021; Kendrick et al., 2014) evaluating the efficacy of interventions for FOF report modest or moderate short-term benefits. Programs incorporating meditation, tai chi, or Pilates appear more effective, particularly when delivered in supervised, group-based formats, compared with home-based programs using written materials, even if tailored, basically because in person program gives direct supervision and immediate feedback, social support and better motivation reducing anxiety while exercising.

Considerable heterogeneity also exists in terms of intervention type, duration (ranging from weeks to 1 year), and outcome measurement tools, further complicating conclusions about the most effective strategies. One review by Zijlstra et al. (2007) reported benefits of home-based programs, which included exercise, nutritional counseling, or home adaptations, compared with controls receiving only general advice and/or social worker visits (Tinetti et al., 1994; Van Haastregt et al., 2000; Yates and Dunnagan, 2001).

In the present study, a multicomponent exercise intervention was delivered in two distinct formats: (1) an onsite, hospital-based, group program supervised 2–4 times per week, and (2) a home-based program supported by the App with daily remote exercise interventions with therapist monitoring. These differences in delivery may partly explain the contrasting results, especially given that more than 50% of App-group participants had no formal education, >50% met criteria for frailty, and >60% were sedentary—factors likely to hinder digital tool use and reduce adherence to remote programs, as reflected in adherence outcomes. Nevertheless, nearly half of App participants achieved >50% adherence and most found it useful. Survey responses regarding the VIVIFIL App indicated that participants generally perceived it as useful, not complex, and well adapted to their activity levels. Nevertheless, results suggest a need to enhance usability regardless of educational background, simplify exercise programs, increase flexibility in goal achievement (e.g., fewer sessions, shorter duration, greater exercise variety tailored to individual needs), and strengthen medium- and long-term adherence through more intensive virtual supervision to reinforce user confidence.

With regard to adherence, The onsite program achieved higher adherence rates at weeks 4, 8, an, with statistically significant differences between groups. Nonetheless, high adherence levels (>50% completion of the program) were maintained by more than 45% of participants in the App group throughout t. Baseline physical activity was the only variable influencing adherence, with sedentary individuals demonstrating the lowest adherence. These findings suggest that onsite interventions promote more sustained engagement and lower dropout rates over time. It should be noted that in the App group, adherence was quantified based on the completion of individual exercises rather than entire sessions, indicating that the lower adherence observed cannot be attributed to stricter tracking criteria but likely reflects differences in participant engagement and supervisionAlmost 70% of participants in the App group reported that the VIVIFIL App was globally useful.

This study has limitations that should be acknowledged. It was a quasi-experimental, non-randomized clinical trial conducted under routine practice, with group assignment determined by hospital resources (one center lacked onsite capacity). Additionally, baseline and week-8 assessments were performed by investigators partly involved in delivering the intervention, and they were not blinded to group allocation, which may have contributed to between-group differences in SFES-I scores Future studies should prioritize a randomized design with blinded outcome assessors to mitigate this potential source of bias. In the same line, the assessment of baseline physical activity level was based on clinical judgment rather than a standardized questionnaire (e.g., the International Physical Activity Questionnaire - IPAQ), which could lead to misclassification. Baseline imbalances in FOF scores limit strict comparability, and results should therefore be interpreted with caution. Nonetheless, analyses emphasized within-group changes and between-group contrasts to strengthen interpretability.

The intervention duration (8 weeks) may be considered short to assess long-term impact on FOF, although evidence suggests that benefits are most pronounced in the short term. Strengths of this trial include the large sample size, advanced age of participants (mean > 80 years), low educational attainment, high prevalence of frailty and sedentary behavior, and comparison of two multicomponent exercise programs of similar structure, which increases the clinical representativeness of findings despite the absence of a non-intervention control group.

Despite the study’s limitations, this is, to the best of our knowledge, the first study directly comparing hospital day-care exercise with a digitally supervised program in very old, frail adults over 80 years.

## 5 Conclusion

FOF is highly prevalent among older adults with a history of falls. An 8-week onsite, group-based multicomponent exercise program significantly reduced FOF and promoted greater short- and medium-term adherence compared with a home-based App-supported program. These results should be interpreted with caution due to the non-randomized design. Although the reduction in FOF was only statistically significant in the onsite group, the App-supported group also showed a meaningful improvement (∼20%), suggesting potential benefits of remotely supervised exercise interventions. Further research is needed to optimize telehealth applications for older adults with FOF, particularly in terms of usability, adherence, and sustained effectiveness.

## Data Availability

The data that support the findings of this study are available on zenodo open Access, cited as: Ruiz-443 Ruiz, L., Neira, M., Huertas-Hoyas, E., Curiel-Regueros, A., García, R., Alonso-Bouzón, C., García de Villa, S., Pilla Barroso, M. J., Secco, F., and Jimenez Ruiz, A. R. (2025). GAIT2CARE: A Database for Evaluating the Effectiveness of Two Exercise Programs in Older Adults using Inertial Gait Analysis and Functional Assessments [Data set]. Zenodo https://doi.org/10.5281/zenodo.15276115.
